# Radiological Findings of Chest X-Rays During the Hajj Seasons 1444–1445 H/2023–2024 G: Diagnostic Quality and Gender Differences in Interpretation Concordance

**DOI:** 10.3390/ijerph22091415

**Published:** 2025-09-11

**Authors:** Ghadah Sulaiman Alsaleh, Abdulaziz Almosabahi, Abdulaziz S. Alhomod, Mohamed Elgaria, Haifa Alharbi, Mohamed Sabry, Mohammed Elttanikhy, Ebtsam Kamal, Hassel Mohammed Alasmary, Khalid Alsuhaibani, Fahad A. Alamri, Reem Hasan, Anas Khan

**Affiliations:** 1 Global Center for Mass Gathering, Ministry of Health, Riyadh 12382, Saudi Arabia; faabalamri@moh.gov.sa (F.A.A.); rhalfifi@moh.gov.sa (R.H.); 2 Radiology Department, Seha Virtual Hospital, Ministry of Health, Riyadh 12382, Saudi Arabiamelgaria@moh.gov.sa (M.E.); halharbi82@moh.gov.sa (H.A.); msselim@moh.gov.sa (M.S.); melttanikhy@moh.gov.sa (M.E.); ebtsamkm@moh.gov.sa (E.K.); halasmary@moh.gov.sa (H.M.A.); kalsuhaibani@moh.gov.sa (K.A.); 3 Emergency Medical Administration, King Fahad Medical City, Riyadh 11525, Saudi Arabia; asalhomod@moh.gov.sa; 4 Innovation Executives Administration, Seha Virtual Hospital, Riyadh 12382, Saudi Arabia

**Keywords:** Hajj, chest X-ray, concordance, gender bias, mass gathering medicine

## Abstract

**Background**: Mass gatherings like the Hajj pilgrimage present unique challenges for radiological services, with high patient volumes and increased respiratory disease risks necessitating reliable chest X-ray interpretation. **Objectives**: The objective of this study is to assess the diagnostic quality, abnormality rates, and peer-review concordance of chest X-rays in patients transferred during the Hajj seasons of 1444–1445 H/2023–2024 G, with an additional focus on gender-based differences in radiological interpretation. **Methods and Materials**: A cross-sectional analysis of 2093 chest X-rays from Hajj healthcare facilities was conducted. Two blinded radiologists independently reinterpreted images using standardized criteria. Data included demographic variables, radiographic findings (quality, opacities, nodules, cardiomegaly, effusions), and tuberculosis likelihood. **Results**: Among interpretable films (89.7% acceptable quality), 69.2% showed abnormalities, primarily opacities (56.4%) and cardiomegaly (27.0%). Tuberculosis was considered probable by radiographic appearance in 21.0% of cases. Peer review demonstrated 94.2% overall concordance. Regression analysis identified the presence of any abnormality (OR = 10.67, *p* < 0.001) and female gender (OR = 2.97, *p* = 0.003) as significant independent predictors of interpretive discordance. A trend towards higher discordance was noted for pulmonary nodules, though it was not statistically significant (9.4% vs. 5.6%, *p* = 0.062). **Conclusions**: While chest X-rays proved reliable for Hajj screening, gender disparities in interpretation and challenges in certain assessments, such as nodule evaluation, highlight opportunities to refine radiological protocols in mass gatherings.

## 1. Introduction

Chest radiography remains a cornerstone of thoracic diagnostic evaluation, offering a rapid assessment of conditions ranging from infectious diseases to chronic cardiopulmonary abnormalities [1]. In high-volume clinical settings, the reliability of radiographic interpretations is critical, as diagnostic accuracy directly impacts therapeutic decisions and public health responses [2]. However, variability in image quality, interpreter expertise, and patient demographics may influence consistency in radiological assessments: a challenge magnified in environments where operational demands strain healthcare systems [3].

The Hajj pilgrimage, as the largest recurring mass gathering, presents a unique natural laboratory to investigate these challenges. It generates a high-volume, demographically diverse dataset of chest X-rays under standardized emergency care conditions where respiratory illnesses dominate morbidity [4,5]. Prior research has established the epidemiology of Hajj-associated pulmonary diseases, but the foundational elements of imaging reliability, specifically, diagnostic quality, overall abnormality rates, and interpreter concordance, remain systematically uncharacterized in this setting [6,7]. Furthermore, the pilgrimage’s gender-segregated care model offers a unique opportunity to examine potential sex-based differences in radiological interpretation with minimized confounding from institutional biases, an area that is critically underexplored.

This study aimed to bridge these evidence gaps through a comprehensive evaluation of chest X-rays obtained during the 1444–1445 H (2023–2024 G) Hajj seasons. It assessed the diagnostic quality, abnormality rates, and peer-review concordance of chest X-rays in patients transferred during these two Hajj seasons, with an additional focus on gender-based differences in radiological interpretation. Our findings will inform quality improvement initiatives for mass gathering medicine, while contributing foundational data on equity in radiological diagnosis. The results hold implications for optimizing imaging protocols in resource-constrained large-scale environments and advancing understanding of demographic influences on diagnostic consistency.

## 2. Methods and Materials

### 2.1. Study Design and Participants

This cross-sectional study was conducted during the Hajj seasons of 1444–1445 H (2023–2024 G). The study examined chest X-ray diagnostic performance and interpretation variability among Hajj pilgrims, with particular attention to gender disparities. Radiology data were collected from all hospitals and primary health centers (PHCs) located in the holy Hajj sites of Makkah, including Mina, Arafat, and Muzdalifah, Saudi Arabia. The study population comprised a total of 2093 patients who underwent chest X-rays during the specified timeframe.

### 2.2. Sample Size

As this study utilized a complete census of all available chest X-rays performed during the 1444–1445 H (2023–2024 G) Hajj seasons across participating facilities in Makkah (Mina, Arafat, and Muzdalifah), no formal sample size calculation was performed. The final cohort of 2093 patients represents the total population of eligible adult pilgrims (aged ≥ 18 years) who underwent chest radiography during the study period and met inclusion criteria.

### 2.3. Inclusion and Exclusion Criteria

The study included adult patients aged 18 years or older who underwent chest X-rays during the Hajj seasons of 1444–1445 H (2023–2024 G). All included cases had available digital chest X-ray images documented within the Hajj electronic health records system across participating healthcare facilities in Makkah, including Mina, Arafat, and Muzdalifah. Patients were excluded if they were younger than 18 years old. Chest X-rays with non-interpretable technical quality (e.g., incomplete lung fields or severe artifacts preventing any assessment) were excluded prior to analysis. Duplicate records or repeated X-rays from the same patient during the study period were excluded, with only the initial scan retained for assessment.

### 2.4. Procedure and Data Collection

Radiology data were systematically retrieved from the Hajj electronic health records (EHR) system immediately following the 1444–1445 H (2023–2024 G) Hajj seasons to ensure completeness. This comprehensive dataset included all chest X-rays performed across health facilities in Makkah (Mina, Makkah, Saudi Arabia, Arafat, Makkah, Saudi Arabia, and Muzdalifah, Makkah, Saudi Arabia), capturing demographic variables (age, gender), radiographic findings (film quality, presence of opacities, nodules, cardiomegaly, mediastinal/hilar abnormalities, effusions), and tuberculosis likelihood assessments.

Two expert radiologists independently reinterpreted all chest X-rays under blind conditions (without access to clinical histories or prior interpretations), using standardized criteria. Prior to the independent review, the radiologists participated in a calibration session to establish unified criteria for acceptable image quality. The agreed-upon standards included proper exposure (allowing for clear visualization of the lung fields and mediastinal structures), the absence of significant rotation or motion artifacts, and complete anatomical coverage from the lung apices to the entire diaphragms. This calibration process was designed to standardize interpretation and enhance the reliability of the readings.

The criteria for diagnosing opacities, nodules, and cardiomegaly were based on the radiologists’ assessment using the following definitions. Opacities were identified as localized areas of increased lung density. Nodules were defined as round/oval-shaped densities larger than 3 mm in the lung fields. Cardiomegaly was evaluated using the cardiothoracic ratio (CTR), with a ratio greater than 0.50 considered indicative of cardiomegaly.

Our study did not utilize a specific standardized radiological scoring system such as the WHO TB CXR classification, but these definitions are widely used in clinical practice.

The assessments (film quality, abnormalities, TB likelihood) were compared for concordance and analyzed by gender and abnormality type. The quality of chest X-rays was assessed based on overall visual clarity, with a focus on identifying positioning errors, exposure problems, and motion artifacts. Original EHR reports were used solely for patient demographics. The collected data were organized and managed using a structured Microsoft Excel spreadsheet. Each variable was clearly defined, and double-entry verification was performed on a subset of records to minimize data entry errors.

### 2.5. Ethical Considerations

This study complied with the ethical principles of the Declaration of Helsinki and received approval from the Institutional Review Board of the Saudi Ministry of Health (IRB Log No: H-02-K-076-0623-967). All patient identifiers (names, medical record numbers) were removed from the extracted radiology reports and demographic data prior to analysis. The de-identified research dataset was securely managed using password-protected Microsoft Excel files stored on a dedicated research computer with restricted physical and electronic access. Only the principal investigator could access the raw data, and no X-ray images or identifiable clinical records were retained in the study database.

### 2.6. Statistical Analysis

Statistical analysis was performed by SPSS version 28 (IBM Co., Armonk, NY, USA). Categorical data were presented as the frequency and percentage and analyzed using the Chi-square test or Fisher’s exact test, as appropriate. Univariate logistic regression analysis was performed to assess the relationship between patients’ gender and peer review. A two-tailed *p*-Value < 0.05 was considered statistically significant.

## 3. Results

The gender of the studied patients is presented in Table 1. The study included a total of 2093 patients, with 1188 (56.8%) males and 905 (43.2%) females.

The chest X-ray results of the studied patients are presented in Table 2. Of 2093 X-rays, 89.7% (95% CI: 8.99–11.79) were of acceptable quality. Among interpretable films (*n* = 1877), 69.2% (95% CI: 65.44–73.02) showed abnormalities, with opacity (56.4%, 95% CI: 53.07–59.92) and cardiomegaly (27.0%, 95% CI: 24.71–29.47) being the most frequent findings. Less common abnormalities included pulmonary nodules (7.4%, 95% CI: 6.18–8.69), mediastinal abnormalities (14.8%, 95% CI: 13.12–16.66), hilar lesions (3.4%, 95% CI: 2.63–4.35), and effusions (18.4%, 95% CI: 16.49–20.43). Tuberculosis was classified as probable in 21.0% (95% CI: 18.97–23.17) of cases.

The peer review of acceptable chest X-ray results of the studied patients is presented in Table 3. Among 1877 acceptable quality X-rays, peer review showed high concordance in interpretation (94.2%), with discordant readings occurring in 5.8% (95% CI: 4.77–7.00) of cases.

**Figure 1:**
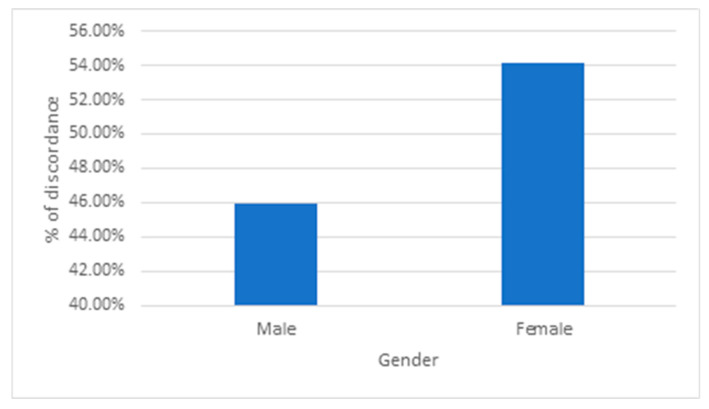
Summary of % of discordance by gender.

The relation between patients’ gender and peer review is presented in Table 4 and Figure 1. Among 1768 concordant peer reviews, 57.4% were male patients and 42.6% were female. In discordant cases (*n* = 109), female patients represented a higher proportion (54.1%) compared to males (45.9%). Female gender was significantly associated with higher odds of discordance (OR = 1.59, 95% CI: 1.08–2.34, *p*-Value = 0.020).

**Figure 2:**
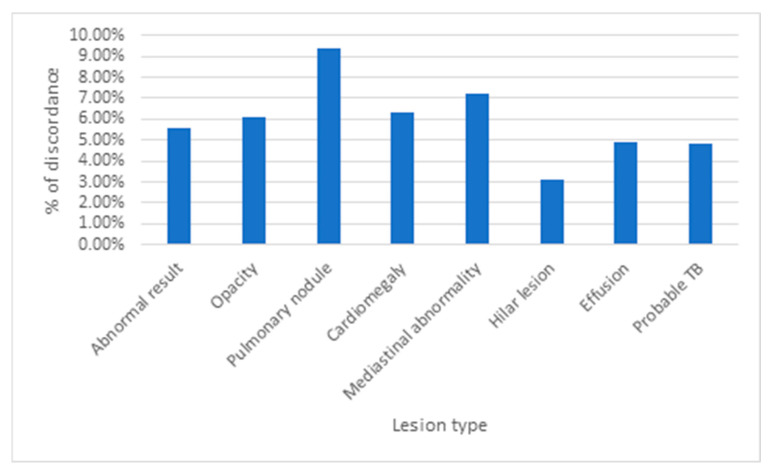
Summary of % of discordance by abnormality type.

The relation between acceptable chest X-ray results of patients and peer review is presented in Table 5 and Figure 2. Peer review concordance rates showed no statistically significant difference between normal (93.8%) and abnormal (94.4%) X-rays (*p*-Value = 0.612). Similarly, no significant associations were found between concordance and specific radiological findings including opacity (*p*-Value = 0.490), cardiomegaly (*p*-Value = 0.572), mediastinal abnormalities (*p*-Value = 0.285), hilar lesions (*p*-Value = 0.350), effusions (*p*-Value = 0.438), or TB likelihood (*p*-Value = 0.353). However, pulmonary nodules demonstrated a non-significant trend toward higher discordance rates (9.4% vs. 5.6%, *p*-Value = 0.062), approaching but not reaching statistical significance.

Multiple logistic regression for the factors associated with the presence of discordance is presented in Table 6. The results showed that the age of the patient is not associated with discordance, while sex and the presence of abnormality are associated. Individual lesions were not included to avoid multicollinearity. The female sex had higher odds of discordance compared to males, OR = 2.97 (95%CI: 1.45 to 6.07), *p*-Value = 0.003. The presence of abnormality in the X-ray is also associated with higher odds of discordance as compared to X-rays with no abnormalities, OR = 10.67 (95%CI: 4.55 to 25.01), *p*-Value < 0.001.

## 4. Discussion

A chest X-ray remains a critical diagnostic tool in mass gathering medicine, particularly during events like the Hajj pilgrimage where respiratory diseases are prevalent [1]. Our findings reveal both the robustness and specific challenges of chest X-ray services under Hajj conditions. The high rate of abnormal studies underscores the clinical value of radiographic screening in this setting.

The predominance of pulmonary opacities was consistent with the expected high burden of respiratory infections and inflammatory conditions in mass gatherings, as demonstrated by Shirah et al. (2017), who identified *Klebsiella pneumoniae* and *Streptococcus pneumoniae* as predominant pathogens in Hajj-related pneumonia cases [4]. This alignment suggests that pulmonary opacities may reflect both acute infections and chronic inflammatory sequelae exacerbated by overcrowding [8,9]. The prevalence of cardiomegaly (27.0%) was surprisingly elevated, suggesting either underlying chronic disease in pilgrims or potential fluid overload from strenuous rituals in extreme heat, indicating a unique cardiovascular burden during Hajj [10]. Of particular public health significance was the 21.0% rate of probable tuberculosis, reinforcing the need for vigilant screening in this international cohort. This rate markedly exceeds the 0.7% undiagnosed TB prevalence reported by Yezli et al. (2023) among symptomatic pilgrims, likely reflecting methodological differences in screening criteria (e.g., our inclusion of radiographic suspicion without microbiological confirmation) [11]. This pattern is consistent with the recent global TB screening literature. For instance, a large-scale study in the Philippines by Marquez et al. (2025) demonstrated the real-world challenge of this approach, where an AI-based chest X-ray screening system achieved a high sensitivity of 95.6%, but a low specificity of 28.1%, resulting in a positive predictive value of only 18.4% [12]. This underscores that while excellent for ruling out disease, a positive X-ray screening often requires confirmation. Similarly, Melendez et al. (2018), in a high-risk screening program in the UK, found that an automated detection system could achieve 95% sensitivity, but at that threshold, specificity was 55.7%, meaning nearly half of the normal studies were flagged as suspicious [13]. Together, these studies confirm that the high rate of radiographic TB suspicion we observed is an inherent characteristic of chest X-ray screening, driven by its high sensitivity but variable specificity, and necessitates subsequent microbiological confirmation to avoid false positives. Notably, Ajlan et al. (2024) found that 57% of TB-screening chest X-rays had technical artifacts, yet only 14% compromised diagnostics [14], implying that our high abnormality detection occurred despite potential technical limitations. The convergence of these studies underscores Hajj as a high-risk setting for TB transmission, particularly among pilgrims with comorbidities or prior TB exposure, as identified by Yezli et al. (2023) [11]. These findings collectively advocate for optimized radiographic protocols and targeted screening for high-risk subgroups, balancing diagnostic sensitivity with resource constraints in mass gatherings. Our results directly inform such strategies. The high volume of abnormal studies supports the use of a chest X-ray as a primary screening tool. However, the high rate of radiographic TB suspicion, coupled with the technical and interpretive challenges identified, argues for a tiered screening protocol. This could involve using chest X-rays for initial triage, followed by rapid molecular testing for patients with radiographic abnormalities suggestive of TB to reduce false positives and prioritize resource-intensive isolation and treatment. This integrated approach would enhance the efficiency and accuracy of TB screening in the demanding Hajj environment, ensuring better protection of public health.

The high overall concordance rate between radiologists demonstrates that chest X-ray interpretation remains largely consistent even under operational pressures, aligning with Kim et al. (2022), who reported an 86.8% concordance between radiologists and a deep-learning algorithm (Lunit INSIGHT CXR) in a multicenter screening cohort [15]. However, the 5.8% discordance rate, while relatively low, warrants attention as it may reflect challenging cases where an additional review could prevent misdiagnosis, a finding echoed by Kromrey et al. (2024), who noted that AI-human discordance often arises in subtle or complex pathologies [16]. Notably, a non-significant trend towards higher discordance was observed for pulmonary nodules (9.4% vs. 5.6% for other findings, *p* = 0.062), likely due to the inherent difficulties in characterizing small nodules on portable radiographs without prior comparisons. This mirrors Kromrey et al.’s (2024) observation that nodules were among the most frequently discordant findings between AI and radiologists, underscoring their diagnostic complexity [16]. In contrast, Al-Rabiah et al. (2025) found no significant differences in emergency vs. radiology residents’ interpretation of critical findings (e.g., pneumothorax), suggesting that discordance may be pathology-specific, rather than universally applicable [17]. The higher inter-radiologist concordance in our study (94.2%) compared to AI-assisted settings (86.8% in Kim et al.) [15] implies that human expertise remains superior in contextual interpretation, though AI could mitigate discordance in resource-limited scenarios. This suggests that targeted double-reading could be considered for challenging findings like nodules in high-stakes settings like Hajj, where portable X-rays’ technical limitations exacerbate diagnostic uncertainty.

The significantly higher discordance rate for female patients raises important questions about potential biological and technical factors. While the OR of 1.59 indicates a statistically significant increase in discordance for female patients, the clinical relevance of this discrepancy warrants careful consideration. However, we did not control for potential confounding variables such as age, comorbidities, imaging site differences, and body habits. These factors may influence radiographic quality and contribute to discordance, particularly in female patients due to anatomical and technical considerations. Furthermore, our study was not designed to adjudicate which reader was ‘correct’ in cases of disagreement, and thus we cannot definitively state whether these discordances led to under-diagnosis or over-diagnosis, or involved primarily minor subjective differences. However, given that the discordance was significant and not isolated to a single finding, it suggests a systematic variation in interpretation that could potentially impact clinical decision-making for a substantial number of female pilgrims. In a high-stakes screening environment like Hajj, where triage decisions must be made rapidly, even a small increase in interpretation variability could have important consequences for equity of care and resource allocation. Therefore, this finding highlights a need for further investigation to determine the nature and clinical impact of these discordant readings. While anatomical differences such as breast tissue superimposition may contribute to interpretation challenges, Weng et al. (2023) demonstrated that cropping breast tissue from images did not resolve performance gaps in AI models, suggesting that physiological differences alone cannot explain gender disparities [18]. This aligns with Glocker et al.’s (2023) findings, where foundation models underperformed for female patients (6.8–7.8% lower accuracy), implicating dataset-specific biases rather than inherent biological factors [19]. Alternatively, this could reflect unconscious bias in evaluating female chest radiographs, as highlighted by the Radiological Society of North America (2023), which reported that AI models trained on non-representative datasets exacerbated sex-based performance disparities [20], a phenomenon that may extend to human interpreters in high-volume settings like Hajj. The persistence of these disparities across both AI and human interpretations (Weng et al., 2023; Glocker et al., 2023) [18,19] challenges the assumption that technical factors (e.g., breast tissue) are the primary drivers, instead pointing to systemic issues in training protocols or diagnostic criteria. This finding requires particular attention, as it suggests current protocols may not equally serve all pilgrims, echoing calls for bias-aware model development (Glocker et al., 2023) and gender-specific calibration of diagnostic thresholds in mass gatherings [19].

The high diagnostic quality of chest X-rays demonstrates the effectiveness of current imaging protocols in Hajj settings, surpassing the 57% technical issue rate reported by Ajlan et al. (2024) in Saudi TB screening radiographs, while aligning with their finding that most artifacts (86%) did not critically compromise interpretation [14]. This strong performance is particularly notable given the challenging field conditions of mass gatherings, contrasting sharply with Ayasrah et al.’s (2025) Jordanian study where only 15% of chest X-rays met all quality standards [21], highlighting the success of Hajj-specific protocols in maintaining diagnostic integrity. The predominance of opacities and nodules among abnormalities suggests these findings should receive particular scrutiny in quality control measures, echoing Hu et al. (2022) recommendation for targeted segmentation algorithms to address region-specific quality issues like lung field loss [22]. The maintained high concordance across most abnormality types (94.4% for abnormal vs. 93.8% for normal, *p* = 0.612) further supports the reliability of radiographic interpretation in this high-volume context, outperforming the 14% diagnostic-compromise rate in the Ajlan et al. cohort despite similar environmental challenges [14]. While the 10.3% suboptimal but interpretable films indicate room for improvement, particularly in minimizing artifacts identified by Hu et al. (2022) as scapula overlap and clavicle unflatness [22], the overwhelming majority of studies met diagnostic standards, validating a chest X-ray as a dependable first-line tool during Hajj when supported by robust quality frameworks like those proposed by Ayasrah et al. (2025) for high-throughput screening environments [21].

Our observed gender distribution (56.8% male) reflects the well-documented Hajj participation pattern where male pilgrims predominate, aligning with the 3:1 male-to-female ratio reported by Shirah et al. (2017) in their analysis of 1059 hospitalized pilgrims [4]. This consistency confirms our cohort’s representativeness of the broader Hajj population, where cultural pilgrimage traditions and travel patterns typically result in higher male attendance. Such demographic fidelity is valuable for clinical research in mass gathering medicine, ensuring our radiological findings reflect the actual composition of the Hajj population served during the pilgrimage season.

The multiple logistic regression analysis provides crucial insight into the drivers of the interpretive discordance observed in our study. The finding that the presence of any radiographic abnormality was the strongest predictor of discordance (OR = 10.67) is highly intuitive. It indicates that the majority of disagreements occurred in complex cases with visible findings, where subjective interpretations of opacities, nodule characteristics, or other subtle features, naturally lead to inter-reader variability. This is further substantiated by the non-significant trend we observed towards higher discordance for nodules. More notably, the female gender was also a significant independent predictor of discordance (OR = 2.97). This result confirms that systematic, non-biological factors are likely to be at play. In contrast, the non-significant result for age confirms that discordance is not a function of the patient’s age but is specifically linked to case complexity and patient gender. Therefore, these results directly identify two key areas for quality improvement: implementing targeted double-reading or adjudication protocols for abnormal studies and developing technical guidelines to optimize image acquisition and interpretation for female pilgrims.

These results collectively paint a picture of a generally reliable but imperfect radiological assessment under mass gathering conditions. The identified gaps, particularly in female patient interpretation and nodule characterization, suggest specific targets for quality improvement initiatives. Future work should focus on standardizing approaches to these challenging scenarios while maintaining the overall high reliability demonstrated across most interpretations. The findings ultimately support continued use of chest radiography in Hajj medical services, with heightened attention to equitable interpretation across patient demographics.

### 4.1. Strengths and Limitations

This study provides the first comprehensive evaluation of chest X-ray diagnostic quality and interpretation concordance during the Hajj pilgrimage, offering unique insights into radiological practices in mass gathering medicine. The large sample size spanning two Hajj seasons enhances the generalizability of findings, while blind independent reviews by expert radiologists strengthen the validity of concordance assessments. The inclusion of gender-based analysis addresses an important gap in understanding potential disparities in radiological interpretation. However, the study is limited by its reliance on retrospective data from electronic health records, which may lack detailed clinical context for some cases. Additionally, while the study identified interpretation discrepancies, it could not determine which readings were clinically more accurate without follow-up diagnostic confirmation. The potential for selection bias must be acknowledged, as our cohort comprised only patients who were clinically referred for X-rays, who likely represent a sicker population than the average pilgrim and may not fully reflect the prevalence of abnormalities in the general Hajj population. Furthermore, the lack of clinical correlation data (e.g., follow-up CT scans, lab results, patient outcomes) means we could not ascertain the clinical significance of the discordant readings. Specifically, the classification of tuberculosis as ‘probable’ was based solely on radiographic findings and lacked microbiological confirmation (e.g., sputum smear, culture, or PCR). This is a significant limitation, and the reported rate of probable TB should be interpreted as radiographic suspicion rather than a confirmed diagnosis. We acknowledge that clinical confirmation through a gold standard such as CT or pathology results was not included in this study. Instead, we focused on inter-radiologist agreement, which is often used in clinical settings where chest X-rays are interpreted in isolation. While this study highlights the consistency between radiologists, incorporating a gold standard for comparison in future research would be valuable for improving the validation of interpretations and reducing potential biases. We recognize that portable X-rays may have limitations in quality, such as image rotation or motion artifacts, which can affect the accuracy of interpretation, particularly in cases like cardiomegaly. As portable chest X-rays are commonly used in clinical settings, this study reflects real-world practice; however, incorporating follow-up imaging (e.g., CT) or clinical validation would reduce the risk of overestimating or misinterpreting findings such as cardiomegaly. Furthermore, while the acceptable quality percentage was based on the radiologists’ overall assessment, a more detailed breakdown of factors such as positioning, exposure, and motion blur would offer valuable insight into the areas requiring focused improvements. We recommend that future studies incorporate these more detailed categories to enhance the understanding of image quality limitations and improve radiographic protocols. Finally, we acknowledge that utilizing standardized scoring systems such as the WHO TB CXR classification could have provided a more structured approach to evaluating opacity and nodule findings. Future studies incorporating such classification systems may enhance the reproducibility and comparability of results, particularly in the context of large-scale screening for diseases such as tuberculosis.

### 4.2. Clinical and Practical Implications

The high rate of abnormal chest X-rays and substantial discordance in gender-based interpretations observed in this study underscore the need for standardized imaging protocols during mass gatherings. These findings advocate for mandatory radiologist calibration sessions before Hajj to improve diagnostic consistency, particularly for female pilgrims whose results showed higher interpretation variability. The implementation of tiered quality control measures for X-ray technicians operating in field conditions should be prioritized, alongside gender-sensitive review processes for critical findings like tuberculosis, given the probable TB rate. For policymakers, the suboptimal but interpretable X-ray rate justifies investments in portable digital radiography systems with AI-assisted quality alerts. Clinically, the frequent detection of cardiomegaly and effusions suggests Hajj medical teams should maintain high suspicions for cardiovascular complications, even in patients presenting with respiratory symptoms.

## 5. Conclusions

This study demonstrates that while chest radiography remains an essential diagnostic tool for Hajj medicine, persistent interpretation disparities, particularly for female pilgrims alongside observed challenges in nodule assessment, reveal systemic challenges in mass gathering radiology. The findings underscore the need to move beyond technical quality improvements and address deeper interpretive biases that may compromise equitable care. It is important to interpret these findings within the study’s limitations, including potential selection bias and the lack of confirmatory clinical data, which prevent conclusions about the clinical impact of interpretation discordance. By acknowledging these limitations alongside the demonstrated reliability of current protocols, this work redefines priorities for radiological services establishing a new benchmark for quality that integrates both diagnostic accuracy and equity considerations, and calls for the implementation of bias-aware, standardized interpretation protocols for mass gatherings.

## 6. Future Recommendations

Further research should prospectively evaluate the impact of AI-based quality control tools on reducing interpretation variability in mass gathering settings. Despite the discussed challenges with AI bias, the integration of AI-assisted tools into Hajj screening holds significant promise. Future implementation must be predicated on the use of debiased AI models trained on diverse, representative datasets and operate within a human-in-the-loop (HITL) model to mitigate identified gender biases and ensure equitable diagnostic accuracy. Studies comparing the diagnostic accuracy of portable versus stationary X-ray systems during Hajj could optimize equipment deployment strategies. Longitudinal assessments of gender disparities in radiological interpretation across diverse clinical contexts would help determine whether observed differences reflect anatomical factors, bias, or disease prevalence variations. Additionally, the development of Hajj-specific radiographic interpretation guidelines, informed by this study’s abnormality prevalence data, could standardize care delivery. Finally, integrating clinical outcome data with radiological findings in future cohorts would strengthen correlations between imaging results and patient prognoses.

## Figures and Tables

**Table 1:** Gender of the studied patients (*n* = 2093).

Item	N	%
**Gender**		
**Male**	1188	56.8
**Female**	905	43.2

**Table 2:** Chest X-ray results of the studied patients.

Item	N	%	95% CI for Rate
**Film quality**	**(*n* = 2093)**		
**Acceptable**	1877	89.7	
**Not acceptable**	216	10.3	8.99 to 11.79
	**(*n* = 1877)**		
**Normality**			
**Normal X-ray**	579	30.8	
**Abnormal X-ray**	1298	69.2	65.44 to 73.02
**Opacity**			
**No**	817	43.5	
**Yes**	1059	56.4	53.07 to 59.92
**N/A**	1	0.1	
**Pulmonary nodule**			
**No**	1729	92.1	
**Yes**	138	7.4	6.18 to 8.69
**N/A**	10	0.5	
**Cardiomegaly**			
**No**	1369	72.9	
**Yes**	507	27.0	24.71 to 29.47
**N/A**	1	0.1	
**Mediastinal abnormality**			
**No**	1598	85.1	
**Yes**	278	14.8	13.12 to 16.66
**N/A**	1	0.1	
**Hilar lesion**			
**No**	1811	96.5	
**Yes**	64	3.4	2.63 to 4.35
**N/A**	2	0.1	
**Effusion**			
**No**	1531	81.6	
**Yes**	345	18.4	16.49 to 20.43
**N/A**	1	0.1	
**Likelihood of TB**			
**Unlikely**	1470	78.3	
**Probable**	394	21.0	18.97 to 23.17
**N/A**	13	0.7	

CI: Confidence interval, TB: Tuberculosis.

**Table 3:** Peer review of acceptable chest X-ray results of the studied patients.

Item	N	%	95% CI for Rate
**Peer review**			
**Concordance**	1768	94.2	
**Discordance**	109	5.8	4.77 to 7.00

CI: Confidence interval.

**Table 4:** Relation between patients’ gender and peer review.

Item	Peer Review		OR (95%CI)	*p*-Value
	Concordance (*n* = 1768)	Discordance (*n* = 109)		
**Gender**				
**Male**	1014 (57.4%)	50 (45.9%)	Ref.	
**Female**	754 (42.6%)	59 (54.1%)	1.59 (1.08 to 2.34)	0.020

Categorical data are presented as frequency (%), OR: Odds ratio, Statistical significance at *p*-Value < 0.05.

**Table 5:** Relation between acceptable chest X-ray results of patients and peer review.

Item	N	Peer Review		*p*-Value
		Concordance	Discordance	
**Normality**				
**Normal**	579	543 (93.8%)	36 (6.2%)	0.612
**Abnormal**	1298	1225 (94.4%)	73 (5.6%)	
**Opacity**				
**No**	817	773 (94.6%)	44 (5.4%)	0.490
**Yes**	1059	994 (93.9%)	65 (6.1%)	
**Pulmonary nodule**				
**No**	1729	1633 (94.4%)	96 (5.6%)	0.062
**Yes**	139	125 (90.6%)	13 (9.4%)	
**Cardiomegaly**				
**No**	1369	1292 (94.4%)	77 (5.6%)	0.572
**Yes**	507	475 (93.7%)	32 (6.3%)	
**Mediastinal abnormality**				
**No**	1598	1509 (94.4%)	89 (5.6%)	0.285
**Yes**	278	258 (92.8%)	20 (7.2%)	
**Hilar lesion**				
**No**	1811	1704 (94.1%)	107 (5.9%)	0.350
**Yes**	64	62 (96.9%)	2 (3.1%)	
**Effusion**				
**No**	1531	1439 (94%)	92 (6%)	0.438
**Yes**	345	328 (95.1%)	17 (4.9%)	
**Likelihood of TB**				
**Unlikely**	1470	1381 (93.9%)	89 (6.1%)	0.353
**Probable**	394	375 (95.2%)	19 (4.8%)	

Categorical data are presented as frequency (%), Statistical significance at *p*-Value < 0.05.

**Table 6:** Multiple logistic regression for factors associated with discordance.

	Odds Ratio	95% CI		*p*-Value
Age (in years)	1.00	0.99	1.02	0.632
Gender (female)	2.97	1.45	6.07	0.003
Having abnormality	10.67	4.55	25.01	<0.001

## Data Availability

The dataset used and/or analyzed during the current study are available from the corresponding author on reasonable request.
